# Treating nonsuicidal self-injury (NSSI) in adolescents: consensus based German guidelines

**DOI:** 10.1186/s13034-016-0134-3

**Published:** 2016-11-29

**Authors:** Paul L. Plener, Romuald Brunner, Jörg M. Fegert, Rebecca C. Groschwitz, Tina In-Albon, Michael Kaess, Nestor D. Kapusta, Franz Resch, Katja Becker

**Affiliations:** 1 Dept. of Child and Adolescent Psychiatry and Psychotherapy, Central Institute of Mental Health, J5, 68159 Mannheim, Germany; 2 Dept. of Child and Adolescent Psychiatry and Psychotherapy, Centre for Psychosocial Medicine, University of Heidelberg, Heidelberg, Germany; 3 Dept. of Child and Adolescent Psychiatry and Psychotherapy, University of Ulm, Ulm, Germany; 4 Dept. of Clinical Child and Adolescent Psychology and Psychotherapy, University of Koblenz-Landau, Landau, Germany; 5 Dept. of Psychoanalysis and Psychotherapy, Medical University of Vienna, Vienna, Austria; 6 Dept. of Child and Adolescent Psychiatry, Psychosomatics and Psychotherapy, University Hospital of Marburg and Philipps-University Marburg, Marburg, Germany

**Keywords:** Nonsuicidal self-injury, NSSI, Self-harm, Adolescents, Guideline, Therapy

## Abstract

**Background:**

Nonsuicidal self-injury (NSSI) is a frequent and clinically relevant phenomenon in adolescence. Within Europe, Germany has one of the highest prevalence rates in youth with lifetime prevalence ranging between 25 and 35%. However, treatment guidelines for NSSI are not yet available.

**Methods:**

Consensus based clinical guidelines were created by a working group consisting of members of eleven medical, psychological or psychotherapeutic professional national associations, and two members of patient self-help and prevention groups. The guidelines were developed in consecutive expert meetings and literature searches and agreed on in a final consensus conference.

**Results:**

Given that evidence on both the psychotherapeutic and psychopharmacological treatment of NSSI is limited, a consensus based approach was chosen. The consensus indicated that due to the accumulating evidence on the efficacy of psychotherapeutic approaches, core elements of psychotherapy should be provided in treatment of NSSI. A specific psychopharmacological therapy of NSSI cannot be recommended. In addition, the guidelines provide recommendations for surgical intervention of NSSI.

**Conclusions:**

In accordance with the heterogeneous level of evidence, recommendations for the clinical management of NSSI in adolescence were made during a consensus conference after reviewing available literature. There is still a lack of knowledge on prevention as well as clinical interventions, which needs to be addressed by further clinically relevant studies.

**Electronic supplementary material:**

The online version of this article (doi:10.1186/s13034-016-0134-3) contains supplementary material, which is available to authorized users.

## Background

Both public and researcher awareness of and interest in nonsuicidal self-injury (NSSI) has increased in recent years, especially since adoption of the term NSSI as a new diagnostic entity in section three of the fifth edition of the diagnostic and statistical manual (DSM 5) [1]. The DSM 5 criteria define NSSI as repetitive (occurring on more than 5 days within 1 year), direct altering body tissue in a non-socially sanctioned manner, and as being undertaken without suicidal intent [1].

In the past, different definitions were used to describe self-injurious behavior with or without suicidal intent, among them terms like “parasuicide”, “self-mutilation” or “self-wounding” [2]. To date, many studies use terms like “Deliberate Self-Harm” (DSH), which includes all forms of self-injurious behavior regardless of its suicidal intent [3], and can best be viewed as an “umbrella term” [4] for self-harming behaviors, also including NSSI and nonsuicidal self-poisoning, which is not captured in NSSI [5]. It somehow resembles the definition of “parasuicide” provided by Power and Spencer [6], who stated that parasuicide “is a variable concept, both in terms of suicidal intent […] and medical lethality” [6, p. 228], whereas it has been argued that terms like para-suicide were “superseded” by “deliberate self-harm” “in recognition that not all episodes involved definite suicidal intent” [7, p. 326].

There is still an ongoing discussion as to whether the inclusion of NSSI in the DSM 5 is warranted or not. On the one hand, creating a category of NSSI can prevent especially adolescents from being automatically “labeled” as having borderline personality disorder, and acknowledges the fact that self-injury can be undertaken without suicidal intent and that consensus-based pathways can be developed for best practice treatment [8, 9]. On the other hand, it has been argued that a clear distinction between non-suicidal and suicidal intent is often not possible and that there can be ambivalence towards the question of suicidal intent [7]. The ongoing debate on these issues is unsettled and it has been stated in a review on the studies using the DSM 5 criteria that some criteria still need clarification [10]. However, the debate about the validity of NSSI has been fruitful, as it created many studies focusing on the association of NSSI and suicidality [11], often describing NSSI as risk factor for suicide attempts [12], preceding them [5, 13]. In comparison to adolescents “only” reporting NSSI, those reporting a combination of NSSI and a history of a suicide attempts reported sexual abuse more often and had higher rates of hospitalizations as well as a more sever history of NSSI [14]. With regards to suicide, it has been shown that the risk of suicide was significantly increased in individuals presenting to an emergency room with self-harm [3] and in individuals with self-cutting in other body areas than arms and wrists [15]. By combining different concepts, Hamza et al. [16] provided an integrated model of NSSI and suicidal behavior, proposing that NSSI predict suicidal behavior (Gateway theory), with the association between NSSI and suicidality being influenced by third variables like BPD (Third Variable Theory) and that NSSI will change the acquired capability to commit suicide.

An increasing amount of literature has identified risk factors for NSSI. By reviewing data from longitudinal studies, female gender, previous NSSI, a history of a suicide attempt or psychiatric disorders were reported most often [17]. Using a meta-analytic approach to identify risk factors for NSSI, previous NSSI, hopelessness, Cluster B symptoms and prior suicidal thoughts or attempts were identified as strongest risk factors [18]. From an inpatient sample of 72 female adolescents alexithymia was identified as risk factor for NSSI according to DSM 5 diagnosis [19]. It seems that NSSI itself is also a risk factor, not only for recurrent NSSI (as stated above), but also for interpersonal stressful life events, with the frequency of NSSI predicting the number of interpersonal stressors among girls [20].

NSSI is a prominent and frequent phenomenon with rates of repetitive and single occurrence around 18% in adolescents from community samples worldwide [21, 22] and self-evident higher rates of around 50% in child and adolescent psychiatric inpatients [23]. A recent Cochrane review focuses on children and adolescents with self-harm, but not specifically with NSSI [24]. Likewise, the National Institute for Health and Care Excellence (NICE) presented a quality standard for the care of patients presenting with self-harm [25]. However, although consensus based approaches on NSSI have been addressed to inform the public about the management of people with self-harm [26], specific guidelines on NSSI for health care providers are still lacking.

Germany has seen especially high rates of NSSI. In community samples of adolescents, lifetime prevalence of single or repetitive NSSI is reported to be between 25 and 35% [27, 28], representing the second highest prevalence rate in Europe [28] and the highest in German speaking countries [29]. It remains unclear, why this is the case, as suicide rates (which had been discussed as an explanation) do not correspond to NSSI rates on a national level [29] and will be a minimum estimate as some of those who would have been recorded as NSSI will have died as an unintended consequence of actions that were high-lethality but of low suicidal intent. These numbers induce an urgent need to provide evidence based guidelines for diagnostic assessment and treatment of NSSI in adolescents. Therefore, the German Society of Child and Adolescent Psychiatry, Psychosomatic and Psychotherapy (DGKJP) set out to coordinate national guidelines on NSSI in adolescents.

## Methods

As literature on the treatment process in NSSI is still limited, a consensus based approach was chosen. This decision was based on the guideline process as proposed by the association of the scientific medical societies (AWMF) in Germany, which is the largest organization coordinating guideline development on a national level.^1^ For the process of guideline development, a group of representatives from professional organizations involved in the treatment of NSSI in adolescents was formed. During initial meetings, this group decided which questions needed to be addressed. To assure coverage of relevant facts from different areas of NSSI intervention and research, different stakeholders (treatment providers, an organization representing patient´s needs, organizations involved with prevention) were part of the group. The balance was checked by the AWMF. Questions were formulated in the group. This was followed by a structured literature search (for example see Additional file 1: Figure S1), leading to diagnostic assessment and treatment recommendations drawn from empirical data if available. If literature was not available, not generalizable to German adolescents, or the level of evidence was low, a consensus was reached based on clinical knowledge (good clinical practice). All recommendations were discussed and voted for in a structured consensus conference at the end of the guideline development process and the level of consensus was documented. All consensus statements had to be voted on with all of them reaching 100% approval finally. The finished guideline was then sent to the boards of professional associations involved in the development of the guideline for final consent and has been approved by all participating professional associations.

The consensus group consisted of members from eleven different national professional organizations from the field of child and adolescent psychiatry, psychology, psychotherapy, pediatrics, and pediatric surgery (see Additional file 2: Figure S2). Furthermore, one representative from an organization providing prevention work and one representative from a self-help organization for people affected by NSSI and suicide were part of the consensus group. All members had to state their financial sources in a formalized way, which was forwarded to the board of the DGKJP to check for potential competing interests.

All clinical recommendations provided below follow a grading pattern provided by the AWMF with three different types of recommendations: “should” (strong recommendation), “ought to” (moderate recommendation) and “may be considered” (optional) [30, 31]. All clinical recommendations and their grading (from strong to optional recommendations) were voted on, reaching consensus in 100%. The following paper will focus on the recommendations provided on both diagnostic assessment, as well as treatment strategies, which were consented in the process of guideline development and are available online in German in its full version [32].

## Results

### Assessment procedures

In clinical contact with adolescents showing NSSI, direct communication is the primary form of exploration and may be supplemented by standardized questionnaires (see Table 1). Although there are several validated questionnaires on NSSI, they are mostly used in a research context. However, they can be of use in complementing information on NSSI, by adding a structured assessment procedure. If possible, adolescents should be interviewed about NSSI without others being present. In addition, taking the history should involve external sources such as family members. Suicidality must be explored and a full mental health assessment should take place, also taking into account comorbid mental disorders. In addition external factors that influence NSSI like family conflicts, bullying, school problems etc. should be explored. In primary contact, the need for immediate medical help needs to be evaluated and status of tetanus vaccination needs to be explored, to assure safety after self-inflicted open wounds. If there is a need for immediate medical intervention (such as surgical intervention), somatic treatment should be prioritized.

**Table 1:** Assessment of NSSI

Basic assessment of NSSI *should* include
As a first step, somatic assessment should be conducted and physical treatment (e.g. surgical dressing) initiated if necessary
Full mental health assessment with a special emphasis on assessing suicidality should be conducted
Frequency and methods of NSSI should be assessed
Factors influencing NSSI both within and outside the family (including school environment, peer group) should be assessed
As part of the psychological assessment of NSSI: specific questionnaires may be considered as support

### Treatment

#### Therapeutic setting

In principal, treatment should primarily take place in an outpatient setting. This is based on the principle of keeping up the highest possible level of functioning and lowering the impact on every day´s life to allow for continuity of family and social relationships. However, outpatient settings should only be used where there is no obvious risk to the individual. Apart from safety concerns, there might be other issues, which could influence a decision towards treatment in an inpatient or day-treatment setting (see Table 2).

**Table 2:** Treatment setting in NSSI

Outpatient treatment of NSSI
Outpatient treatment *should* be initiated if the psychological, social, and academic (or ability to work) level of functioning is sufficient, an ability to cooperate is existent and criteria for inpatient treatment are not met
Inpatient treatment of NSSI
Acutely suicidal patients *must* be treated in an inpatient setting at a Department of Child and Adolescent Psychiatry
*Should* be initiated in case of severe bodily harm or situations making close supervision necessary
*Ought to* be initiated if the environment is detrimental to treatment success
*May be considered*, if there is no possibility of achieving sufficient diagnostic assessment in an outpatient setting
*May be considered* if no outpatient treatment is available
*May be considered* if no sufficient treatment response is achieved in an outpatient or day treatment setting

#### Surgical treatment

It is possible that individuals with NSSI may require surgical treatment (e.g. stitches), which should be carried out in a professional and neutral manner (without judgmental comments). Whenever other professions are involved, trustful cooperation and sharing of information between professions (based on the informed consent of caregivers and assent of the minor) is crucial (see Table 3). A multidisciplinary approach in the surgical treatment of patients with NSI has also been put forward by authors from the field of surgery, also stating the necessity of a satisfactory esthetic outcome [33].

**Table 3:** Surgical treatment of NSSI

In the surgical treatment of NSSI
The best possible functional and cosmetic result *should* be strived for
The intervention *should* be made as painless as possible
Emotional responses and judgmental comments *should* be forborne
When the patient is receiving treatment outside of child and adolescent psychiatry, she/he *should* be supervised as long as the potential for imminent threat has not been assessed
A child and adolescent consultation *should* be requested as soon as possible
For patients already in therapy, contact with the therapist *ought to* be sought

#### Psychotherapy

To date, no randomized controlled trials specifically focusing on the treatment of NSSI have been published. Although the number of controlled studies on psychotherapeutic interventions in NSSI is still limited, evidence on the efficacy of psychotherapy in the treatment of NSSI accumulates [34, 35]. In a recent systematic review Calear et al. [36] analyzed seven interventions in DSH, with three studies describing a significant decrease of DSH after the intervention, three studies describing no effect and one study reporting a significant effect in favor of the control condition. In another recent meta-analysis and meta-regression including both data from studies in adults and adolescents, no evidence for an efficacy for NSSI was reported with the exception of mentalization based treatment (MBT) [37]. On the other hand, the recent cochrane review on psychosocial interventions for self-harm in adults concluded that treatment effects for self-harm were observed for cognitive behavioral therapy (CBT), group-based-emotion-regulation-psychotherapy, mentalization and dialectical behavior therapy (DBT) [38]. Looking into literature on adolescents, reduction of self-harm has been shown in a randomized controlled trial of Dialectical Behavior Therapy for Adolescents (DBT-A) [39]. Evidence for the reduction of self-harm in treatment studies has also been provided in studies on cognitive behavioral therapy (CBT) [40], cognitive analytic therapy (CAT) [41], and mentalization based treatment for adolescents (MBT-A) [42]. However, none of the included trials exclusively focus on NSSI. A randomized, controlled study of CBT in Germany [43], specifically focusing on NSSI, has recently been finished and is awaiting analysis and publication.

When pooling data from several therapeutic studies, there is a clear signal that psychotherapy is effective in reducing NSSI, however without a clear trend for any therapeutic approaches being superior [34]. Starting from this background, core elements of psychotherapy which can be applied throughout different therapeutic approaches in the treatment of NSSI in adolescents were recommended (see Table 4).

**Table 4:** Core-elements in the psychotherapeutic treatment of NSSI

The following core elements of the psychotherapeutic treatment of NSSI *should* be considered
Clear contracts in the case of suicidality or NSSI
Building commitment for treatment
Psychoeducation
Identifying factors that trigger or maintain NSSI
Providing alternative behavioral skills or problem solving strategies in the case of NSSI
Attention to and treatment of comorbid psychiatric disorders as suggested in evidence based guidelines

When planning treatment for NSSI, comorbid psychiatric disorders should always be assessed. In every individual case, it should be considered whether the treatment of NSSI, or the treatment of the comorbid disorder should initially be in the focus of the therapy. Comorbid disorders should be treated according to corresponding guidelines.

#### Psychopharmacology

Suggestions on psychopharmacological treatment are based on the understanding that psychopharmacological treatment of NSSI is not to be used as a single treatment strategy and needs to be put in the context of a broader multimodal, psychotherapeutic treatment frame. Overall, studies are lacking on the psychopharmacological treatment of NSSI, especially concerning adolescents [44]. A larger body of literature is available for the treatment of self-injury, especially in patients with BPD. The available Cochrane review on pharmacological treatment in BPD however reports that non of the available studies showed a significant effect on self-injury [45]. In addition, a more recent Cochrane review on the pharmacological treatment of self-harm in adults, reports a lack of data, thus not allowing conclusions to be drawn on the efficacy of pharmacological interventions [46].

In adolescents, a specific psychopharmacological therapy of NSSI cannot be recommended. Nevertheless, the need for an acute psychopharmacological intervention in the case of severe inner tension (with an urge to self-injure) emerges at times. In this case, pharmacological sedation can be used where other approaches have proven unsuccessful. As higher rates of NSSI in those receiving benzodiazepines have been reported from the TORDIA study [47] and another study reported a lack of efficacy in treating NSSI with these compounds [48], the use of benzodiazepines should be restricted to clearly defined cases and weighing the risk–benefit ratio on an individual level. This should include evaluation of the setting, as, for example, the effects of benzodiazepines can be more easily monitored and controlled within an inpatient setting. Lower potency conventional antipsychotics may be administered where preferred and if they are tolerated by the patients. Therefore, the decision on pharmacological treatment is based on personal variables, potential interactions with other drugs and the setting.

**Fig. 1:**
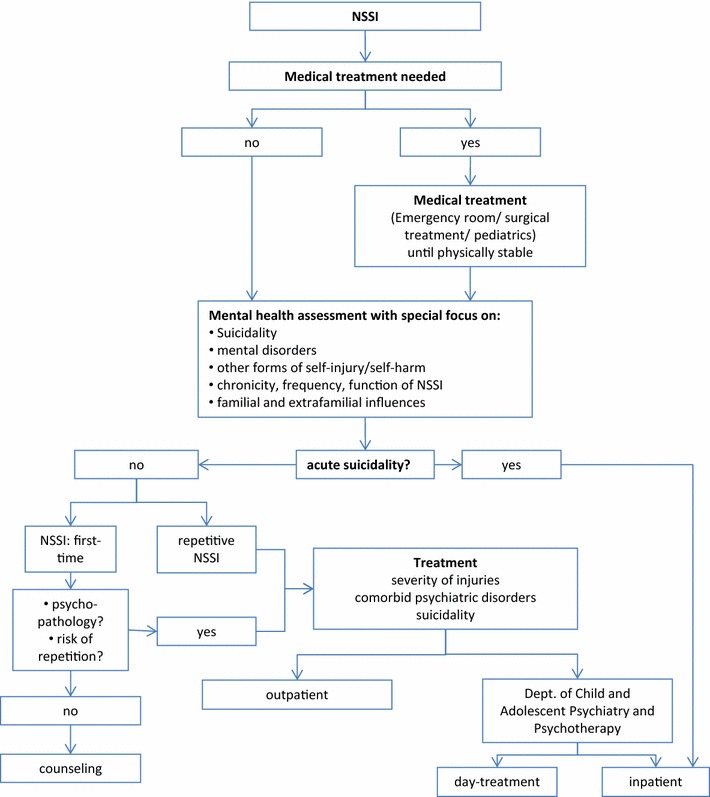
Treatment algorithm for NSSI

Recommendations were finally put together to describe a clinical treatment pathway, as outlined in an algorithm (see Fig. 1). However, these recommendations are not evidence-based due to the lack of data in this field.

#### Prevention

With regards to prevention, the guidelines state that due to a current lack of available studies, the efficacy of prevention programs cannot be commented. This statement still holds through, despite the rapid development of prevention programs in the sector of self-harm. In one study, implementing a school-level prevention program for NSSI was shown not to cause any iatrogenic effects, however the rate of help-seeking in this population was unchanged [49]. In the large European SEYLE program, no effects of the different preventive strategies employed were seen on NSSI, whereas a significant reduction in suicide attempts and ideation was reported from a Youth Aware of Mental Health Programme [50], findings that were also observed in other prevention trials [36]. Up to date there is, to the best of our knowledge, still no prevention program showing an effect in preventing NSSI.

## Discussion

To the best of our knowledge, these guidelines on NSSI present the first approach to provide treatment recommendations for NSSI for healthcare professionals. These recommendations are based on a consensus of professional organizations representing several key-players in the treatment of NSSI. Following a structured consensus process, national guidelines were developed. As the importance of NSSI is increasing—with high prevalence rates in adolescents coupled with the inclusion of NSSI in Section three of DSM 5—knowledge about best-practice models of NSSI are urgently needed.

So far, there have been some approaches trying to provide guidelines for the treatment of self-harm. Kelly and colleagues [26] provided mental health first aid guidelines for the treatment of “deliberate nonsuicidal self-injury” (comparable to a NSSI definition), based on a consensus process involving 26 professionals, 16 people, who had engaged in NSSI and three people providing care for people with NSSI. After defining deliberate self-injury and describing different methods, the guideline provides recommendations about how to approach self-injuring individuals and when mental healthcare should be obtained. As these guidelines’ focus is on mental health first aid, their advice is for the general public, they do not, therefore, provide details on optimal treatment choice or administration. Following this line of recommendations, the NICE quality standard for self-harm [25] also focus on the first response to self-harm in the initial assessment as well as on the prevention of its recurrence. It has to be kept in mind that as the NICE quality statement´s focus is on self-harm, it also includes reactions to behavior undertaken without or with suicidal intent. The NICE statement proposes that all patients should be offered psychosocial and risk assessment. This should include “physical health, mental state, safeguarding concerns, social circumstances and risk of further self-harm or suicide” [25, Statement 2]. Comprehensive psychosocial assessments should identify “personal factors that might explain an act of self-harm” [25, Statement 3] and should be provided by a “healthcare professional” [25, Statement 3]. It is also made clear that people, who have self-harmed should be monitored in the healthcare setting to reduce the risk of self-harm [25 Statement 4]. Regarding assessment, all of these recommendations are comparable to those suggested in the here presented German consensus guidelines on NSSI. The list of quality statements as taken from the NICE quality standards [25] is fully in line and allows many cross-comparisons to the German consensus based guidelines, with regards to statements about assessment and monitoring for individuals presenting with self-harm. Recommendations about further therapeutic interventions are stated in a sense that people with self-harm should have a “discussion with their lead healthcare professional about the potential benefits of psychological interventions specifically structured for people who self-harm” and a plan on how to gain support during the transition between services should be collaboratively developed between the individual presenting with self-harm and the healthcare professional [25, Statement 7]. It is stated clearly that service providers should ensure mechanisms to provide “3–12 sessions of a psychological intervention specifically structured for people who self-harm” [25, Statement 7]. Statements on psychosocial interventions are in line with the German consensus guideline presented here and are—beyond that—more specific in pointing out that a short-term intervention is potentially beneficial to patients presenting with self-harm.

Recently, a Cochrane review has been published focusing on interventions for self-harm in children and adolescents [46], including eleven trials with 1126 participants. All included studies reported data on psychosocial trials, none of pharmacological trials. Authors stated that therapeutic assessment increased adherence to treatment, whereas it did not affect the repetition of self-harm. While many specific treatments failed to show an effect on the repetition of self-harm, MBT-A as well as DBT-A were able to show a reduction of the frequency of self-harm. The authors concluded that results from studies on therapeutic assessment, MBT-A, DBT-A and CBT warrant further studies [46]. In a recent meta-analysis, Calati and Courtet [37] found no evidence for the effectiveness of psychotherapeutic treatments other than MBT in the treatment of NSSI, whereas a recent Cochrane Review on self-harm in adults identified several psychotherapeutic approaches to be effective in reducing self harm [38]. As to date there is no clear-cut evidence for the superiority of a specific psychotherapeutic approach in treating NSSI in adolescents, the guideline group decided on an integrative approach. In line with previous recommendations, the present guidelines for NSSI recommend core elements of psychotherapy, e.g. psychoeducation, identifying triggers and maintaining factors and providing alternative skills.

Guideline development did identify several gaps in the literature. It may well be that the proposed DSM 5 definition provides a chance to use a uniform definition, which will increase comparability between studies. This will also serve future research on the neurobiology of NSSI, as the suggested diagnostic criteria lend itself to be explored using a Research Domain Criteria approach [51]. Given the high prevalence of NSSI in adolescents, further treatment studies are urgently needed. This should also include stepped-care approaches to support different sub-groups of adolescents who self-injure. Furthermore, research on children and adolescents who self-injure, living outside their families is lacking. As also pointed out by the recent Cochrane review [46], no RCTs on the psychopharmacological treatment with NSSI as the primary outcome are available. Therefore, recommendations on psychopharmacological treatment are based on individual treatment experiences, case reports as well as studies using self-harm or NSSI as secondary outcomes, often not specific for the age-group of children and adolescents.

## Conclusions

In conclusion, there is an overlap between recommendations on the treatment of self-harm as proposed by the NICE guidelines and the recommendations provided by the consensus based German NSSI guidelines presented here. Furthermore, the recommendations are in line with a recent systematic review on the treatment literature concerning children and adolescents, who self-harm [46]. Despite the fact that the new German NSSI guidelines are focusing on NSSI specifically and suicidality will be targeted in a separate upcoming guideline, there seems to be a joint understanding over and about different nomenclatorial boundaries (NSSI vs. self-harm). It needs to be borne in mind that suicidality and NSSI are not mutually exclusive, thus assessment of suicidal ideation/behavior before, during or after NSSI should be the gold standard [52].

The guideline provides a new set of consensus based recommendations that can be used to support the development of new interventions to aid professionals in the management of children and adolescents in great need of support. To the best of our knowledge, this is the first treatment guideline for the recently created diagnostic entity of NSSI in the DSM 5.

## Additional files

**Additional file 1: Figure S1.** Example for literature search.

**Additional file 2: Figure S2.** Professional associations involved in guideline development.

## Abbreviations

DSM 5: diagnostic and statistical manual, fifth edition
DSH: deliberate self-harm
NICE: National Institute for Health and Care Excellence
NSSI: nonsuicidal self-injury
AWMF: Arbeitsgemeinschaft Wissenschaftlich Medizinischer Fachgesellschaften: German association of the scientific medical societies
DBT-A: dialectical behavior therapy for adolescents
CBT: cognitive behavioral therapy
CAT: cognitive analytic therapy
MBT-A: mentalization based treatment for adolescents

## Footnotes

1
http://www.awmf.org/leitlinien/awmf-regelwerk/ll-entwicklung/awmf-regelwerk-01-planung-und-organisation/po-stufenklassifikation/klassifikation-s2e-und-s2k.html.
