# Supplementary material for: Treating nonsuicidal self-injury (NSSI) in adolescents: consensus based German guidelines
Source: Child Adolesc Psychiatry Ment Health. 2016 Nov 29;10:46. doi: 10.1186/s13034-016-0134-3 (PMC5126819; doi:10.1186/s13034-016-0134-3)
Supplement: Supplementary file 1 — Additional file 1: Figure S1. Example for literature search. [file 13034_2016_134_MOESM1_ESM.docx]

Search question: Are psychopharmacological drugs effective in reducing NSSI in children and adolescents (up to 18 years of age)?

Searched databases: Medline, Pubmed, EMBASE, PsychINFO

Filter: <18 years, humans

No time limits

Search terms:

Self-injur* OR NSSI OR nonsuicidal self-injur* OR non-suicidal self-injur* OR self-harm OR deliberate self-harm OR DSH OR self-mutil* AND psychopharm* OR antidepress* OR antipsychot* OR neurolept* OR sedative* OR SSRI OR Benzodiazepine* OR SNRI OR mood stabiliz* OR medication
