# Supplementary material for: Treating nonsuicidal self-injury (NSSI) in adolescents: consensus based German guidelines
Source: Child Adolesc Psychiatry Ment Health. 2016 Nov 29;10:46. doi: 10.1186/s13034-016-0134-3 (PMC5126819; doi:10.1186/s13034-016-0134-3)
Supplement: Supplementary file 2 — Additional file 2: Figure S2. Professional associations involved in guideline development. [file 13034_2016_134_MOESM2_ESM.docx]

Angehörige um Suizid e.V.

Berufsverband der Kinder- und Jugendärzte e.V.

Berufsverband für Kinder- und Jugendpsychiatrie, Psychosomatik und Psychotherapie in Deutschland e. V.

Bundesarbeitsgemeinschaft der Leitenden Klinikärzte für Kinder- und Jugendpsychiatrie, Psychosomatik und Psychotherapie e. V.

Bundespsychotherapeutenkammer

Deutsche Gesellschaft für Kinderchirurgie

Deutsche Gesellschaft für Kinder- und Jugendmedizin

Deutsche Gesellschaft für Kinder- und Jugendpsychiatrie, Psychosomatik und Psychotherapie

Deutsche Gesellschaft für Psychologie

Deutsche Gesellschaft für Suizidprävention

Deutsche Gesellschaft für Verhaltenstherapie

Freunde fürs Leben e.V.

Nationales Suizidpräventionsprogramm
